# Expanded roles of pyruvate-sensing PdhR in transcription regulation of the *Escherichia coli* K-12 genome: fatty acid catabolism and cell motility

**DOI:** 10.1099/mgen.0.000442

**Published:** 2020-09-25

**Authors:** Takumi Anzai, Sousuke Imamura, Akira Ishihama, Tomohiro Shimada

**Affiliations:** ^1^​ School of Agriculture, Meiji University, Kawasaki, Kanagawa, Japan; ^2^​ Laboratory for Chemistry and Life Science, Institute of Innovative Research, Tokyo Institute of Technology, Yokohama, Kanagawa, Japan; ^3^​ Micro-Nanotechnology Research Center, Hosei University, Koganei, Tokyo, Japan

**Keywords:** PdhR, *Escherichia coli*, gSELEX, transcription regulation, fatty acid β-oxidation, cell motility, pyruvate

## Abstract

The transcription factor PdhR has been recognized as the master regulator of the pyruvate catabolism pathway in *Escherichia coli *, including both NAD-linked oxidative decarboxylation of pyruvate to acetyl-CoA by PDHc (pyruvate dehydrogenase complex) and respiratory electron transport of NADH to oxygen by Ndh-CyoABCD enzymes. To identify the whole set of regulatory targets under the control of pyruvate-sensing PdhR, we performed genomic SELEX (gSELEX) screening *in vitro*. A total of 35 PdhR-binding sites were identified along the *E. coli* K-12 genome, including previously identified targets. Possible involvement of PdhR in regulation of the newly identified target genes was analysed in detail by gel shift assay, RT-qPCR and Northern blot analysis. The results indicated the participation of PdhR in positive regulation of fatty acid degradation genes and negative regulation of cell mobility genes. In fact, GC analysis indicated an increase in free fatty acids in the mutant lacking PdhR. We propose that PdhR is a bifunctional global regulator for control of a total of 16–23 targets, including not only the genes involved in central carbon metabolism but also some genes for the surrounding pyruvate-sensing cellular pathways such as fatty acid degradation and flagella formation. The activity of PdhR is controlled by pyruvate, the key node between a wide variety of metabolic pathways, including generation of metabolic energy and cell building blocks.

## Data Summary

gSELEX data for PdhR have been deposited in the ‘Transcription factor profiling of *Escherichia coli *’ (TEC) database at the National Institute of Genetics (https://shigen.nig.ac.jp/ecoli/tec/). The authors confirm all supporting data, code and protocols have been provided within the article or through supplementary data files.

**Impact Statement**

Pyruvate is a key node on the metabolic map, and its homeostasis is strictly controlled in the cell. Transcription factor PdhR senses the intracellular pool of pyruvate in *Escherichia coli *, and serves as the master regulator of the pyruvate catabolism pathway, including the oxidative decarboxylation of pyruvate to acetyl-CoA by the pyruvate dehydrogenase complex and the respiratory electron transport system. By using the gSELEX-chip method, we have identified about 20 genes or operons as novel regulatory targets of PdhR, including a number of cellular pathways all connected with the key node pyruvate, such as fatty acid degradation and cell motility control.

## Introduction

PdhR, a GntR-family transcription factor of *Escherichia coli,* was originally identified as a repressor of the *pdhR-aceEF-lpd* operon, which encodes the E1 (AceE), E2 (AceF) and E3 (Lpd) subunits of the pyruvate dehydrogenase complex (PDHc) and PdhR itself [1]. PdhR monitors the intracellular pool of pyruvate, and thereby controls the expression level of PDHc [2]. Because the PdhR-pyruvate complex is unable to bind target DNA, the *pdhR-aceEF-lpd* operon is derepressed in the presence of high concentrations of pyruvate. PDHc plays a key role in central carbon metabolism (CCM) through controlling the interconnection between glycolysis and the tricarboxylic acid (TCA) cycle. We have previously identified two regulatory targets of PdhR, *ndh* encoding NADH dehydrogenase II and *cyoABCDE* encoding the cytochrome *bo*-type oxidase, by using the genomic SELEX cloning and sequencing (gSELEX-clos) method [3]. These two newly identified targets together form the terminal electron transport system of the respiratory pathway downstream of the PDH cycle [3]. In addition, two regulatory targets, the glycolate utilization *glcDEFGBA* operon and the *dcw-cluster* for interlink between the energy generation system and the replication machinery, were identified based on transcriptome analysis of a *pdhR* mutant and PdhR-overproducing strain [4]. Altogether, a total of nine targets are listed in RegulonDB ([5, 6], regulondb.ccg.unam.mx).

Pyruvate, the terminal product of glycolysis, plays a key role in connecting a variety of metabolic pathways [7, 8]. Pyruvate forms a node between a number of metabolic pathways, including the TCA cycle, gluconeogenesis, fatty acid metabolism and amino acid metabolism. In response to stressful environmental conditions, pyruvate participates as a scavenger of reactive oxygen species. The total number of hitherto identified PdhR targets is, however, only nine as listed in RegulonDB ([5, 6], regulondb.ccg.unam.mx). Attempts have been made to identify the whole set of regulatory targets for PdhR. For this, we employed the gSELEX-chip screening system with use of a tiling array [9, 10]. In this system, test transcription factor (TF)-bound DNA segments were affinity-isolated with a purified His-tagged TF. gSELEX analysis allows identification of target promoters, genes and operons under the direct control of each TF (see Discussion for the merit of *in vitro* screening). The PdhR-bound DNA probes were analysed via the gSELEX-chip system using an *E. coli* K-12 tiling array. A total of 35 PdhR-binding sites were identified along the *E. coli* K-12 genome. Possible involvement of PdhR in the regulation of the newly identified candidate targets was analysed in detail by gel shift assay, RT-qPCR and Northern blot analysis. The results indicated the participation of PdhR in regulating the genes for fatty acid degradation and cell motility.

## Results

### Search for the regulatory targets of PdhR

To gain insight into genome regulation by PdhR in *E. coli,* we tried to identify the whole set of target promoters, genes and operons under the control of PdhR. For this, we employed gSELEX screening, in which purified His-tagged PdhR was mixed with a collection of *E. coli* K-12 W3110 genome fragments of 200–300 bp in length, and PdhR-bound DNA fragments were affinity purified using Ni-NTA agarose for the identification of PdhR recognition sequences [10]. This gSELEX screening was repeated for up to five cycles. The original mixture of genome DNA fragments formed smeared bands on PAGE, but after repeated gSELEX screening, the PdhR-bound DNA formed sharper bands on PAGE, indicating the enrichment of specific DNA fragments with specific binding activity to PdhR. Previously, we identified two regulatory targets, *ndh* encoding NADH dehydrogenase II and *cyoABCDE* encoding the cytochrome *bo*-type oxidase, by using the gSELEX-clos method [3]. These two newly identified targets together form the terminal electron transport system of the respiratory pathway downstream of the PDH cycle [3].

**Fig. 1.**
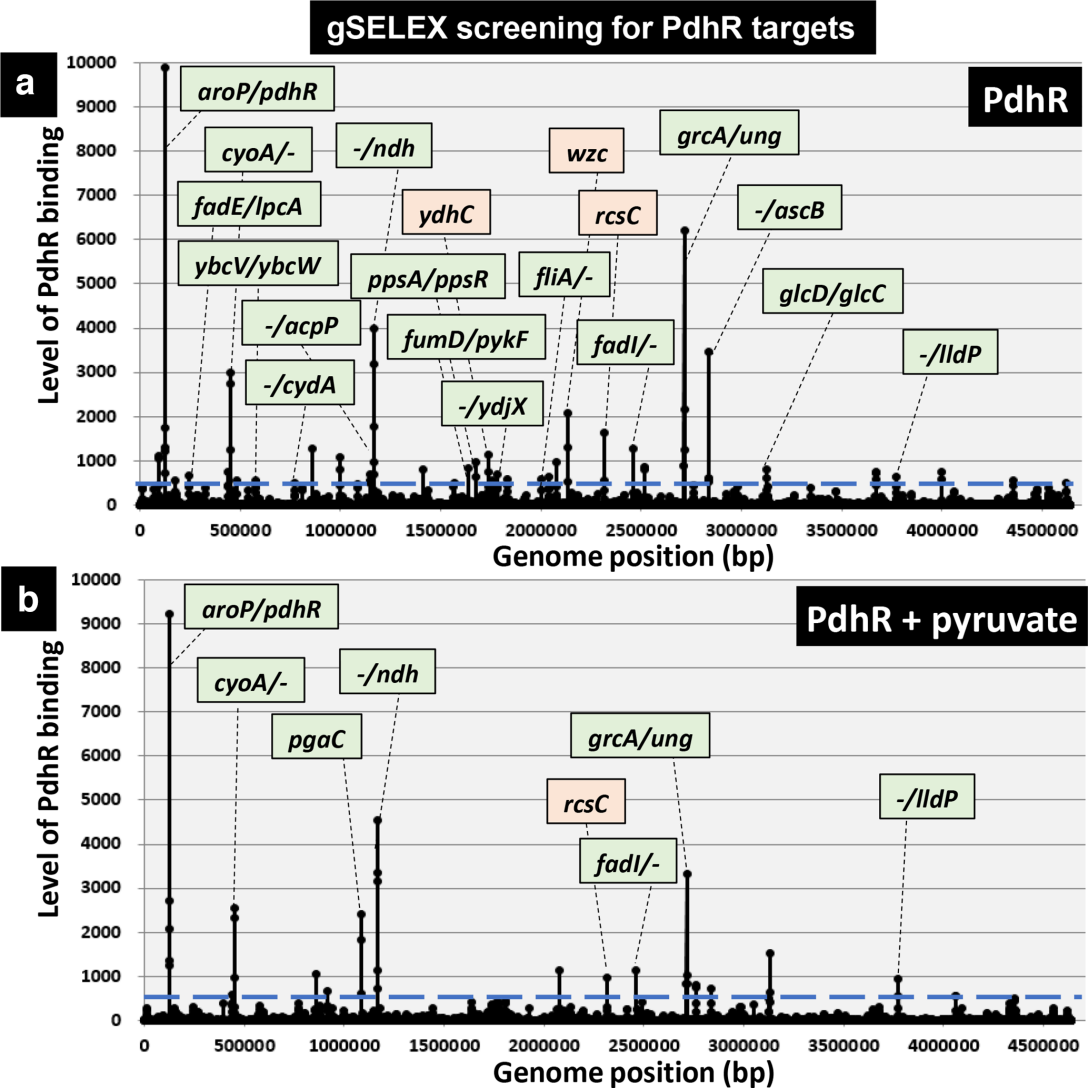
Identification of PdhR-binding sites on the *E. coli* K-12 genome by gSELEX-chip analysis. Genomic SELEX screening of PdhR-binding sequences was performed in the absence (a) or presence (b) of pyruvate. After gSELEX, a collection of DNA fragments was subjected to gSELEX-chip analysis using the tiling array of the *E. coli* K-12 genome. The *y*-axis represents the relative number of PdhR-bound DNA fragments, and the *x*-axis represents the position on the *E. coli* genome. The regulation target genes of PdhR at high-level peaks are shown along the *E. coli* genome. Peaks shown in green represent the type-A and type-B PdhR-binding sites inside spacer regions, while peaks shown in orange represent the type-D PdhR-binding sites inside ORFs (for typing see the legend to Table 1). The cut-off level of 500 is shown by a blue line, and the list of all PdhR-binding sites by setting this cut-off level is given in Table 1.

To identify the whole set of regulatory targets of PdhR, we employed the gSELEX-chip (tiling array chip) system using an *E. coli* tiling array containing a total of 43 450 probes. In the absence of effector, we identified a total of 35 high-intensity peaks by setting the cut-off level above an intensity of 500 (Fig. 1a, Table 1). Of these 35 high-level binding peaks, seven PdhR-binding sites were located within spacers of bidirectional transcription units (Table 1, type-A), and nine PdhR-binding sites were located inside spacers upstream of one ORF but downstream of another ORF (Table 1, type-B). Taking these results together, we predicted the total number of regulatory targets of PdhR is between 16 (7 type-A plus 9 type-B) and 23 (7×2 type-A plus 9×1 type-B).

**Table 1.** PdhR-binding sites on the *E. coli* genome

**Peak position (bp)**	**Intensity (PdhR+** **pyruvate)**	**Intensity (PdhR alone)**	**Function**	**Operon**	**Gene**	**D**	**PdhR site**	**D**	**Gene**	**Operon**	**Function**	**Consensus sequence (PdhR box: AATTGGTnnnACCAATT)**	**Conservation**	**Genome position (bp)**	**Data base**
**Type-A: PdhR-binding sites are located upstream of both of divergentry transcribed genes (total, 7 sites)**															
121966	9 889	9 234	aromatic amino acid:H+symporter	*aroP*	***aroP***	<		>	***pdhR***	*pdhR-aceEF-lpd*	regulator of pyruvate dehydrogenase	AATTGGTaagACCAATT	14/14	122052	***pdhR***
243432	655	290	acyl-CoA dehydrogenase	*fadE*	***fadE***	<		>	***lpcA***	*lpcA*	d-sedoheptulose 7-phosphate isomerase	tccTGGTcatAgCAccT	8/14	243459	–
578844	556	239	DLP12 ; DUF1398 domain-containing protein	*ybcV*	***ybcV***	<		>	***ybcW***	*ybcW*	DLP12 prophage; uncharacterized protein	AtcTGGTctgACCtcTT	10/14	578887	–
1753972	555	352	fumarase D	*fumD*	***fumD***	<		>	***pykF***	*pykF*	pyruvate kinase I	AgcTGGTcagACCttTa	9/14	1753987	–
1785030	679	251	phosphoenolpyruvate synthetase	*ppsA*	***ppsA***	<		>	***ppsR***	*ppsR*	PEP synthetase regulatory protein	AgTTGGTtatACCAAag	11/14	1785098	–
2714448	6 197	3 323	stress-induced alternate pyruvate formate-lyase	*grcA*	***grcA***	<		>	***ung***	*ung*	uracil-DNA glycosylase	AATTGGTaaaACCAtTg	12/14	2714529	***grcA***
3126270	795	488	glycolate dehydrogenase	*glcDEFGBA*	***glcD***	<		>	***glcC***	*glcC*	DNA-binding transcriptional dual regulator	AATTGGTcctACCtgTg	11/14	3126271	***glcD***
**Type-B: PdhR-binding sites are located upstream of single specific genes (total, 9 sites)**															
450964	2 995	2 315	cytochrome bo_3_ ubiquinol oxidase subunit 2	*cyoABCDE*	***cyoA***	<		<	*ampG*			AATTGGTataACCAATg	13/14	451054	*cyoA*
770852	509	390			*mngB*	>		>	***cydA***	*cydAB*	cytochrome bd-I ubiquinol oxidase	tAaaGaTatgACCAAgT	9/14	770832	–
1150668	697	180			*fabG*	>		>	***acpP***	*acpP-fabF*	acyl carrier protein	AcaTGGTctgACCgctt	8/14	1150625	–
1165072	3 993	4550			*ycfP*	>		>	***ndh***	*ndh*	NADH:quinone oxidoreductase II	ttTTGGTatgACCAATg	11/14	1165180	***ndh***
1831272	573	99			*xthA*	>		>	***ydjX***	*ydjXYZABCD*	DedA family protein	–			–
2000362	584	87	RNA polymerase, sigma 28 (sigma F) factor	*fliAZ*	***fliA***	<		<	*fliC*			AAaTGGTaaaACCAcgg	10/14	2000414	–
2458466	1 266	1 122	3-ketoacyl-CoA thiolase	*fadIJ*	***fadI***	<		<	*yfcZ*			cAgcGGTaaaACCtgac	7/14	2458479	–
2839166	3 469	383			*ascF*	>		>	***ascB***	*ascB*	6-phospho-β-glucosidase	cAaaGGTctgACCAcTg	9/14	2839100	–
3775254	632	943			*yibL*	>		>	***lldP***	*lldPDR*	lactate/glycolate:H^+^ symporter	AATTGGccctACCAATT	13/14	3775215	–
**Type-D: PdhR-binding sites with in open reading frames (total, 19 sites)**															
92666	1 113	114			*ftsL*	>	*ftsI*	>	*murE*	*ftsLI-murEF-mraY-murD-ftsW-murGC-ddIB-ftsQAZ-lpxC*	UDP-N-acetylmuramoyl-l-alanyl-d-glutamate—2,6-diaminopimelate ligase	tAaTGGTaacACCAtTa	10/14	92682	–
173132	557	74			*fhuD*	>	*fhuB*	<	*hemL*			–			–
435948	755	584			*thiL*	>	*pgpA*	<	*yajO*			AAagGaTatgACCAtTT	10/14	435814	–
480770	541	16	Hha toxicity modulator	*tomB-hha*	*tomB*	<	*acrB*	<	*acrA*			AAcgGaatcgACCAgcT	8/14	480940	tomB
856236	1 265	1 063	l,d-transpeptidase	*ldtB*	*ldtB*	<	*ybiT*	<	*ybiU*			AAaTGaTctgACCgtgT	9/14	856381	–
999430	1 083	103			*elfD*	>	*elfC*	>	*elfG*	*elfDCG-ycbUVF*	putative fimbrial-like adhesin protein	AAaTGGTtacACCAtTT	12/14	999352	–
1412370	795	21	Rac prophage; endodeoxyribonuclease toxin	*recET-ralR-rcbA-xisR-intR*	*ralR*	<	*recT*	<	*recE*			–			–
1635148	820	407			*ynfO*	>	*ydfO*	<	*gnsB*			AAcaGGTcagACCAgaT	10/14	1635002	–
1676572	978	128	pyridine nucleotide transhydrogenase	*pntAB*	*pntA*	<	*ydgH*	>	*ydgI*	*ydgI-folM*	putative arginine:ornithine antiporter	–			–
1738666	1142	321	LysR family DNA-binding transcriptional regulator	*ydhB*	*ydhB*	<	ydhC	>	*cfa*	*cfa*	cyclopropane fatty acyl phospholipid synthase	ggaTGGTctgACCttTa	8/14	1738515	–
2037932	629	65			*yedX*	>	*msrP*	>	*msrQ*	*msrPQ*	periplasmic prot-l-methionine sulfoxide reductase	AAgTGGcaaaACCAtTg	10/14	2037865	–
2075930	976	1 124			*yeeU*	>	*yeeV*	>	*yeeW*	*yeeRST-cbeA-cbtA-yeeW*	CP4-44 prophage; uncharacterized protein	–			–
2131938	2 066	174	colanic acid biosynthesis glycosyl transferase	*wcaAB*	*wcaA*	<	*wzc*	<	*wzb*			AATTGGTaaaACCttTg	11/14	2132058	–
2317232	1 633	968			*rcsB*	>	*rcsC*	>	*atoS*	*atoSC*	sensory histidine kinase	tcagGGTatgACCAtTT	9/14	2317144	–
2517448	851	74			*yfeD*	>	*gltX*	>	*valU*	*valU*	tRNA-Val(UAC)	tgcgGGTcttACCAATT	10/14	2517346	–
3673834	735	230			*yhjD*	>	*yhjE*	<	*yhjG*			–			–
3997946	740	12			*yigB*	>	*uvrD*	<	*yigE*			–			–
4357756	552	484	lysine decarboxylase 1	*cadA*	*cadA*	<	*cadB*	<	*cadC*			tAgTGGTtaaACggcTT	9/14	4357618	–
4616836	509	216			*deoC*	>	*deoA*	>	*deoB*	*deoBD*	phosphopentomutase	cgTTaGccacgCCAAca	7/14	4616931	–

Typing of gSELEX peaks in ‘Peak position column’ followed the proposal in Ishihama et al. [12] and in TEC database (www.shigen.nig.jp/ecoli/tec/). Type-C (binding site is located spacer downstream of both transcription units) was not identified in the gSELEX pattern of PdhR. PdhRbinding sites indicated in green (type-A and type-B) and orange (type-D) are shown in Fig. 1. Column D indicates the direction of transcription. The genes shown in bold indicate the regulatory targets of PdhR.

In the presence of 1 mM pyruvate, the effector of PdhR, most of the high-intensity peaks were detected even though the peak intensities decreased markedly (Fig. 1b), indicating that the binding affinity of PdhR to the target sequences decreased after interaction with the effector pyruvate, supporting the regulation model of PdhR action through interaction with pyruvate [3]. As the gSELEX screening was successfully used for identification of the whole set of regulatory targets of PdhR, we focused on predicted novel targets for detailed analysis for experimental confirmation.

### Confirmation of PdhR-binding to the newly identified targets

**Fig. 2.**
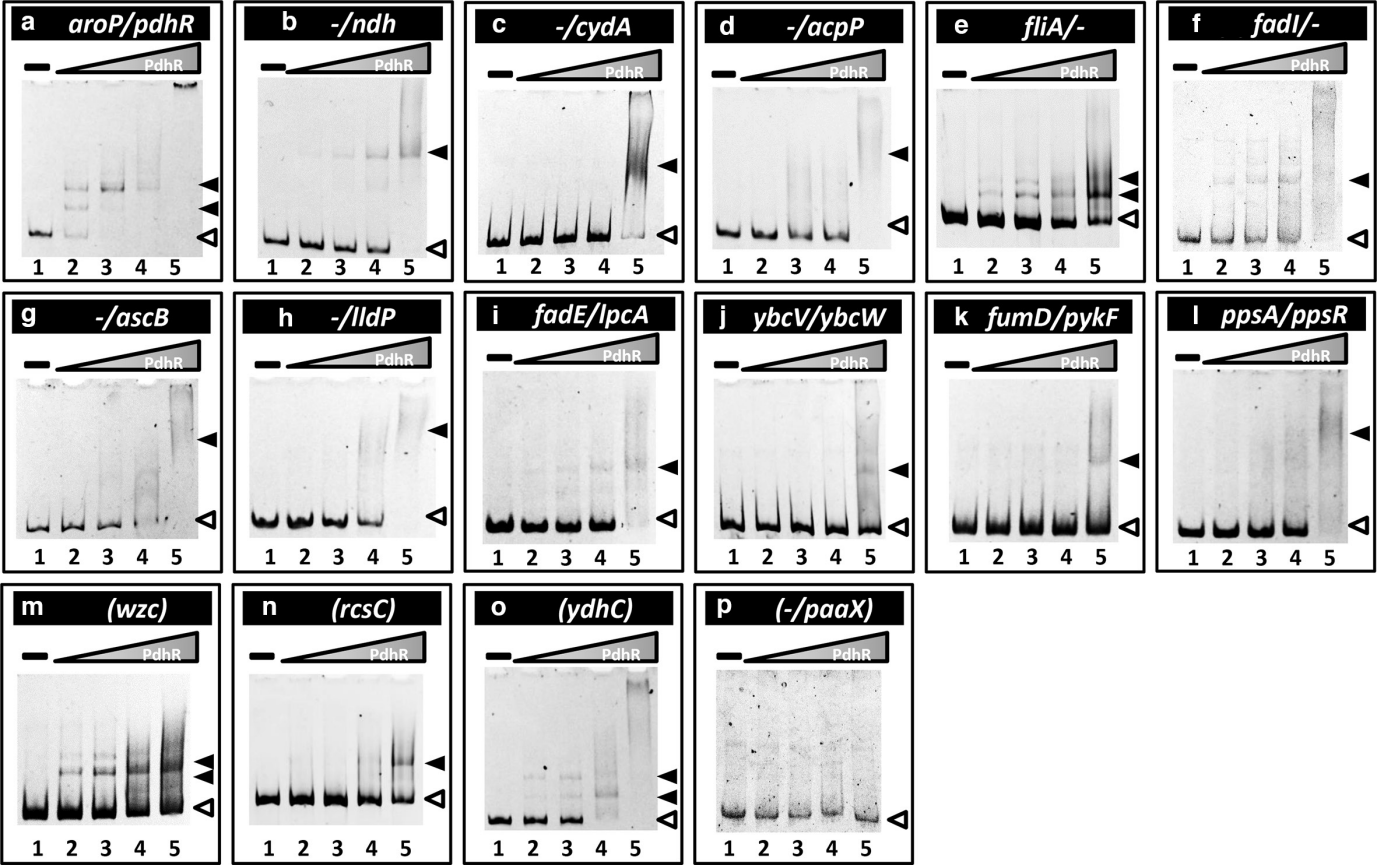
Confirmation of the PdhR-binding *in vitro* to the regulatory targets: gel shift assay. Purified His-tagged PdhR was mixed with 0.2 pmol of each DNA probe corresponding to the PdhR-binding regions shown in Fig. 1. PdhR (pmol) was added: lane 1, 0; lane 2, 1.25; lane 3, 2.5; lane 4, 5.0; lane 5, 10.0. Filled triangles indicate the PdhR–DNA probe complex while open triangles indicate free probe.

To confirm the binding activity *in vitro* of PdhR to the target sites predicted based by the gSELEX screening, we carried out a gel shift assay for detection of PdhR–target DNA complexes. To check the accuracy of the assay system, two DNA probes were prepared for two known targets of PdhR, *pdhR* and *ndh*. Each of these reference probes was mixed with increasing concentrations of purified PdhR and the probe–PdhR mixtures were then directly subjected to PAGE. As expected, these two probes formed PdhR concentration-dependent PdhR–DNA complexes (Fig. 2a, b). Next, ten probes were prepared for the newly identified targets from type-A and type-B (see Table 1) and subjected to the gel shift assay under the same conditions. All ten DNA probes formed PdhR concentration-dependent PdhR–probe DNA complexes (Fig. 2c–l). The binding of PdhR to these targets was indicated as judged based on the disappearance of free probes, but the migration pattern of expected PdhR–probe DNA complexes was variable: some formed clear complex bands (e.g. *fliA* probe for Fig. 2e; *fumD/pykF* probe for Fig. 2k); and some showed smear on gel which is indicative of dissociation of the PdhR–probe complexes during gel electrophoresis (e.g. *fadI* for Sig.2F; and *ppsA/ppsR* for Fig. 2l). The binding was not observed with a non-specific *paaX* promoter region used as an internal reference (Fig. 2p). To test the PdhR-binding activity within the ORF for type-D targets, PdhR-binding was examined for three representative targets, *wzc, rcsC* and *ydhC*. All three targets formed clear shift bands (Fig. 2m–o), indicating PdhR-binding activity to these sites in agreement with their detection by gSELEX screening. TF-binding to ORFs has been observed with only a specific set of TFs [11, 12], implying as yet unidentified regulatory roles for TF-binding inside ORFs.

### Consensus recognition sequence of PdhR

**Fig. 3.**
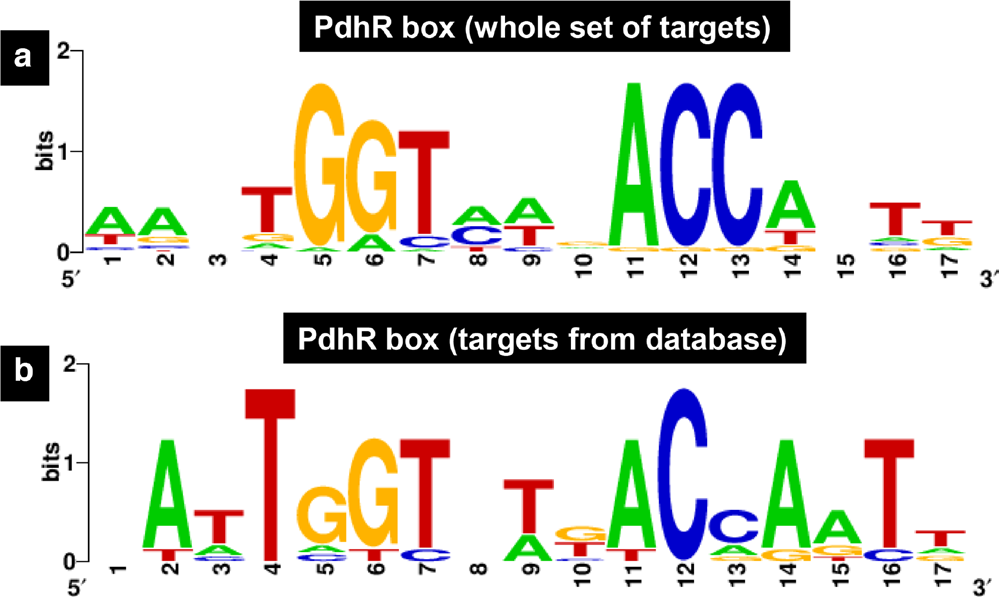
Consensus sequence of the PdhR-box. Sequences of all the probes with high-level binding of PdhR were analysed using dminda 2.0 (http://bmbl.sdstate.edu/DMINDA2/) (see Table 1). (a) Sequences built with the PdhR-target sequences listed in RegulonDB. (b) weblogo (http:// weblogo.berkeley.edu/logo.cgi) was used for matrix construction.

Using the DNA-binding sequence information from a small number of PdhR targets, a 15- or 17-bp-long palindromic sequence consisting of (A)ATTGGTnnnACCAAT(T) was proposed as the consensus recognition sequence of PdhR [2, 3] (Fig. 3b). As we obtained at least 3.9-fold more PdhR-binding sites by gSELEX (35 gSELEX targets versus nine targets listed in RegulonDB), the consensus sequence of PdhR binding was re-evaluated using the whole set of 35 targets including six known targets (see Table 1). A collection of 500-bp sequences from these targets was analysed using the dminda 2.0 program, which was successfully employed for the identification of the PlaR (regulator of plant utilization)-box sequence [13]. We identified a 17-bp-long sequence (Fig. 3a), which contained a complete palindromic trinucleotide sequence GGTnnnACC. The PdhR-binding to neighbouring sites, detected in the previous estimation (Fig. 3b), almost disappeared. Thus, we concluded that this highly conserved 9-bp-long PdhR-box sequence is needed for tight binding of PdhR.

### Transcription regulation *in vivo* of predicted target genes by PdhR

**Fig. 4.**
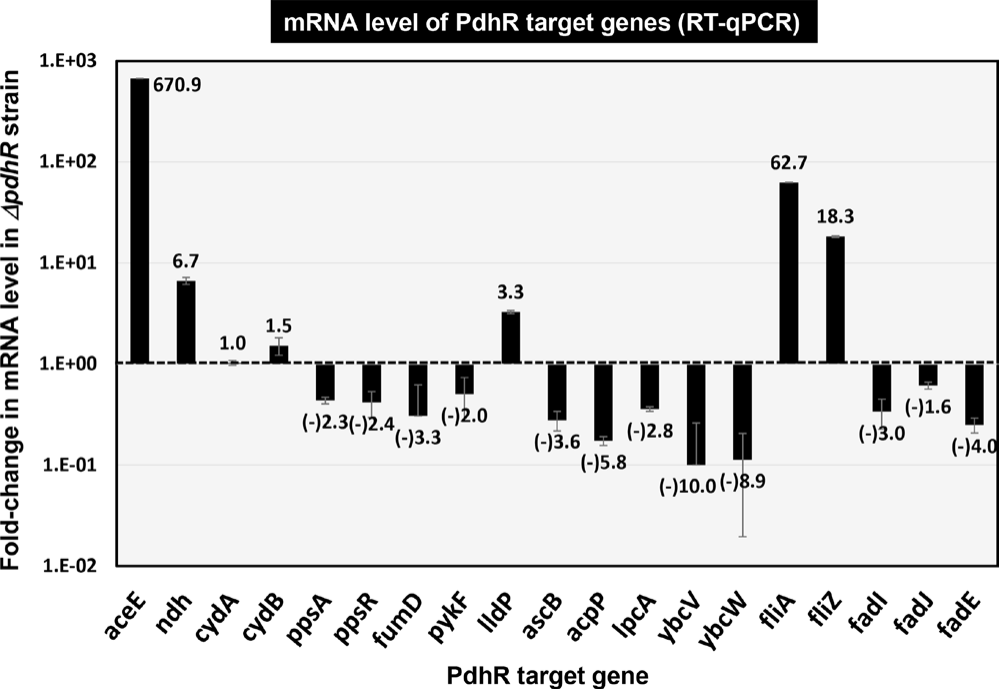
Influence of *pdhR* deletion on transcription of the newly identified PdhR targets by RT-qPCR. *E. coli* wild-type BW25113 and the *pdhR*-deleted mutant JW0109 were grown in M9 medium with 0.2 % succinate. Total RNA was prepared from both the wild-type and *pdhR* mutant, and subjected to RT-qPCR analysis (see Experimental procedures). RT-qPCR was repeated three times and the average means are shown. This list shows mRNA levels of the indicated regulation target genes of PdhR. The *y*-axis represents the relative level of mRNA of each PdhR target gene between wild-type and the *pdhR* mutant, by setting the ratio of 16S rRNA as an internal control between wild-type and the *phdR* mutant.

To examine the regulation *in vivo* of newly identified target genes by PdhR, we next performed RT-qPCR analysis for four groups: 12 genes (*aceE, ndh, cydA, cydB, ppsA, ppsR, fumD, pykF, lldP, ascB*, *acpP* and *lpcA*) for carbon metabolism; three genes (*fadI, fadJ* and *fadE*) for fatty acid β-oxidation; two genes (*fliA* and *fliZ*) for motility and flagellar synthesis; and two genes of unknown function (*ybcV and ybcW*). Total RNA was prepared from exponential-phase cells of wild-type *E. coli* K-12 and its *pdhR*-deleted mutant, and the level of mRNA was measured for these 19 representative target genes. The results for the three groups of the genes with known functions are described below. Regarding the hitherto uncharacterized *ybcV* and *ybcW* genes, the mRNA level decreased 10.0- and 8.9-fold, respectively (Fig. 4), implying the activation of these genes by PdhR. The physiological roles of YbcV and YgcW, however, await further study.

### Regulation of the newly identified targets for carbon metabolism

PdhR was originally identified as the regulator of genes encoding the PDH complex, which catalyses NAD-linked decarboxylation of pyruvate [1]. Later we identified the participation of PdhR in the regulation of *ndh* and *cyoABCDE*, which are involved in respiratory electron transport of NADH, the pathway downstream of the PDH cycle [3]. After repeated RT-qPCR, mRNAs of two known PdhR targets, *aceE* (pyruvate dehydrogenase E1 subunit) and *ndh* (quinone oxidoreductase II), were found to increase in the *pdhR*-defective mutant (Fig. 4), in agreement with the proposed repressor function of PdhR on these two known targets [3]. Here we identified a total of 12 genes as possible targets of PdhR, which are all involved in the CCM, TCA cycle and respiratory electron transfer systems (for details see Discussion). PDHc converts pyruvate and NAD^+^ to acetyl-CoA and NADH, thereby connecting glycolysis and the TCA cycle and generating the redox energy as NADH for aerobic respiration [14]. PdhR was also suggested to regulate the enzymes for producing pyruvate as well as the enzymes utilizing pyruvate as a substrate: LldP (lactate/glycolate: H^+^ symporter) and LldD (lactate dehydrogenase) that together uptake lactate/glycolate and convert them to pyruvate. mRNA for the *lldPRD* operon increased 3.3-fold in the *pdhR* mutant, implying repression of this lactate pathway by PdhR. PykF (pyruvate kinase) converts phosphoenolpyruvate (PEP) to pyruvate, and PpsA (phosphoenolpyruvate synthase) converts pyruvate to phosphoenolpyruvate. *pykF* and *ppsA* mRNAs decreased more than 2-fold in the *pdhR* mutant (Fig. 4), indicating a positive role of PdhR in expression of these genes. *cydAB*, encoding the cytochrome *bd*-type oxidase for electron transport from NADH to oxygen, was not affected by depletion of PdhR under the conditions used here. The mRNA level of *fumD* (fumarase), *ascB* (6-phospho-β-glucosidase), *acpP* (acyl carrier protein) and *lpcA* (d-sedoheptulose 7-phosphate isomerase) decreased in the *pdhR* mutant, suggesting a positive role of PdhR for expression of these genes involved in carbon metabolism.

### Regulation of the newly identified targets for motility

The gSELEX search indicated possible involvement of PdhR in regulation of the *fliAZ* operon, which includes the genes for two key regulators of cell motility. More than 50 genes for flagella and chemotaxis are organized in the coordinately regulated flagella regulon [15]. Transcription of these flagellar-regulon genes forms a highly organized three-stage cascade [15, 16]. The second-stage *fliAZ* operon is under control of the first-stage FlhDC, the master regulator of this hierarchy. FliA is the minor sigma factor RpoF, which controls a set of genes involved in motility and flagellar synthesis [17]. FliZ is another global regulator that plays a role in the regulation of curli expression and the motility system [18]. FliZ antagonizes RpoS-dependent expression of another set of genes needed for transition from exponential growth during the stationary phase [18]. RT-qPCR showed that *fliA* (*rpoF*) mRNA increased 62.7-fold in the *pdhR* mutant (Fig. 4). Accordingly, two representative RpoF-dependent genes, *fliC* (flagellar filament structural protein) and *flgN* (flagellar biosynthesis protein), were found to increase 37.4±0.6- and 19.5±0.1-fold, respectively, in the *pdhR* mutant. RT-qPCR showed a 18.3-fold increase of *fliZ* mRNA in the *pdhR* mutant (Fig. 4).

**Fig. 5.**
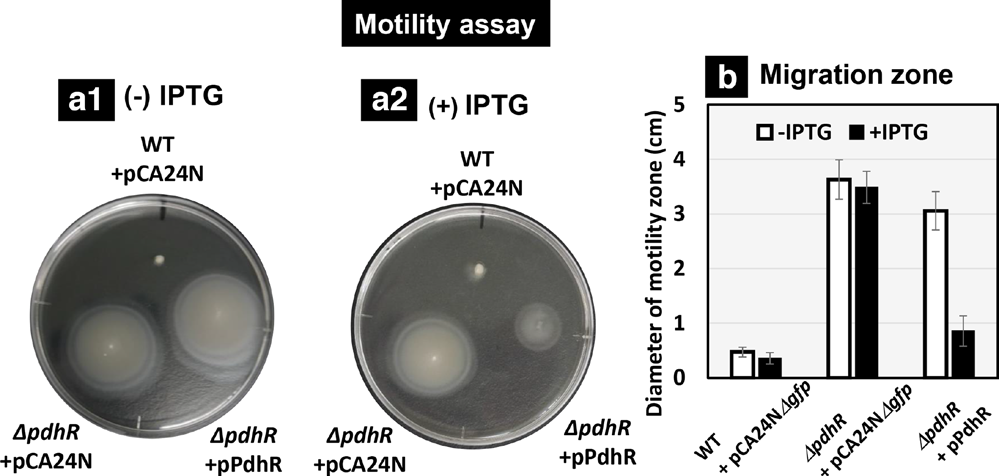
Motility assay on soft agar plates. Transformants of *E. coli* wild-type BW25113, *pdhR*-deleted mutant JW0109 with PdhR expression plasmid or its control empty vector pCA24N*Δgfp* were inoculated on the 0.25 % agar plate in M9 minimal medium with 0.2 % succinate in the absence (a1) or presence (a2) of 50 µM IPTG. After 24 h of incubation, the plate image was observed (a) and the colony diameter was measured as the migration zone (b).

Thus, the *fliAZ* operon was indicated to be under repression by PdhR, indicating a model in which FliA (RpoF) and FliZ together participate in control of planktonic growth. To confirm this hypothesis, we subjected the *pdhR*-defective mutant to the cell motility assay by measuring swimming on 0.25 % soft agar plates in the same medium as employed for mRNA measurement by RT-qPCR. As expected, the *pdhR* mutant showed enhanced swimming compared to the wild-type (Fig. 5a, b). To confirm the direct effect of PdhR, the swimming assay was performed for the same *pdhR* mutant but in the presence of PdhR expression in *trans* using an IPTG-inducible PdhR expression plasmid. The enhanced swimming in the *pdhR* mutant was found to be suppressed after expression of PdhR (Fig. 5a, b). These results support the prediction that PdhR represses flagellar synthesis and motility.

### Regulation of the newly identified targets for fatty acid degradation

Here we also identified possible involvement of PdhR in the regulation of a group of the genes for fatty acid degradation. *E. coli* K-12 utilizes exogenously supplied fatty acids as carbon and energy sources. Fatty acids are the most abundant carbon source, and thus the regulation of fatty acid degradation is important for understanding the regulation of carbon source metabolism. Fatty acids are converted through several steps down to acetyl-CoA, which is then catabolized via the β-oxidation pathway (for details see Discussion). *E. coli* K-12 has two gene sets for the β-oxidation pathway, one consisting of *fadA*, *fadB* and *fadE*, and another composed of the functional homologues *fadI, fadJ* and *fadE*. The FadAB pathway is the main catabolic route under aerobic growth conditions while the FadIJ pathway acts as an alternative route [19]. Fatty acid degradation (β-oxidation) takes place through four recurring steps: (step-1), acetyl-CoA oxidation by FadE; (step-2), 2,3-enoyl-CoA hydration by FadB or FadJ; (step-3), 3-hydroxyacyl-CoA oxidation by FadA or FadJ; and (step-4), 3-ketoacyl-CoA thiolysis by FadI [19]. RT-qPCR showed that mRNA levels of *fadI, fadJ* and *fadE* were decreased 1.6- to 4.0-fold in the *pdhR* mutant (Fig. 4). Thus, we predicted that the genes for a set of fatty acid β-oxidation enzymes are under the positive control of PdhR.

**Fig. 6.**
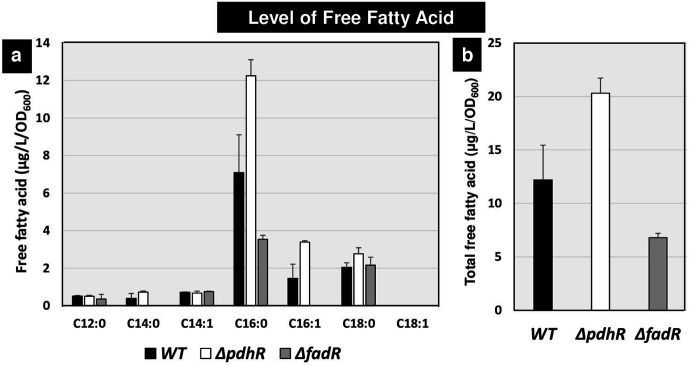
Amount of free fatty acids: individual fatty acids and total fatty acids. *E. coli* wild-type BW25113, *pdhR*-deleted mutant JW0109 and *fadR*-deleted mutant JW1176 were grown in M9 minimal medium with 0.2 % succinate. Total free fatty acids were extracted from exponentially growing cells, and purified by TLC. The amount of each fatty acid was analysed with GC-FID (a). The total amount of free fatty acids was calculated as the sum of individual fatty acids (b). The *y*-axis (μg l^−1^/OD_600_) represents the amount of free fatty acid. The levels of fatty acids in three *E. coli* strains are shown separately: black, wild-type; white, *pdhR* mutant; grey, *fadR* mutant.

To confirm the regulatory role of PdhR in fatty acid degradation, attempts were made to directly measure the intracellular level of fatty acids. *E. coli* K-12 BW25113 was grown in M9–0.2% succinate medium under aeration and whole cell fatty acids were extracted with chloroform/methanol and fractionated by TLC. Each of the free fatty acid spots was subjected to GC-FID (flame ionization detector) analysis. Except for palmitic acid (C16 : 0), the fatty acid contents were essentially the same between the wild-type, the mutant lacking PdhR, and the mutant FadR, the main regulator of the genes for fatty acid β-oxidation [20] (Fig. 6a). The fatty acid level was highest for palmitic acid (C16 : 0) followed by stearic acid (C18 : 0) (Fig. 6a). The levels of both palmitic acid (C16 : 0) and stearic acid (C18 : 0) were, however, higher for the *pdhR*-defective strain. Based on the level of the most abundant fatty acids, the total amount of free fatty acids calculated from the sum of all free fatty acids was 12.2 µg l^−1^/OD_600_ in the wild-type strain (Fig. 6b), but increased to 20.3 µg l^−1^/OD_600_ in the *pdhR* mutant, indicating the activation role of PdhR in fatty acid degradation. On the other hand, the level of total fatty acids, 6.8 µg l^−1^/OD_600_, in the mutant lacking the known repressor FadR of fatty acid degradation was lower than in the wild-type strain, in good agreement with a previous report [21]. Taken together, we conclude that PdhR activates fatty acid β-oxidation.

**Fig. 7.**
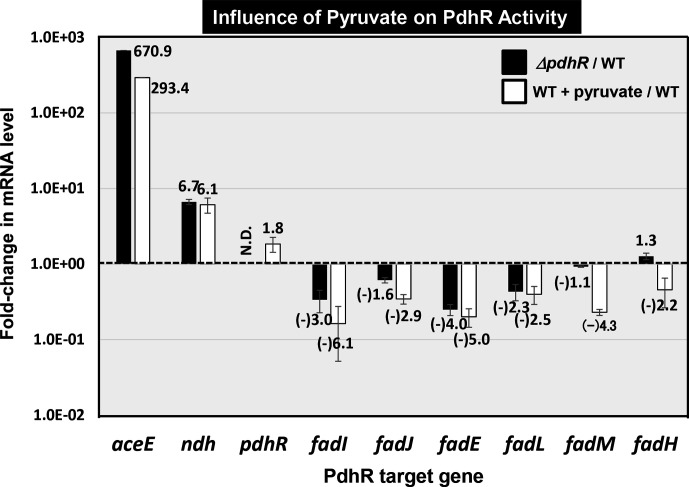
Influence of *pdhR* deletion and pyruvate addition on transcription of the newly identified PdhR targets for fatty acid degradation. *E. coli* wild-type BW25113 and the *pdhR*-deleted mutant JW0109 were grown in M9 minimal medium with 0.2 % succinate in the absence and presence of 3 mM pyruvate. The relative level of mRNA of each PdhR target gene between wild-type and *pdhR* mutant is shown by black bars, while the relative level of mRNA in wild-type *E. coli* in the presence and absence of pyruvate is shown by white bars. The experiment was repeated three times and the average values are shown.

The metabolic steps under the control of PdhR are all related to those involved in catabolism of carbon sources and energy generation, and are interconnected with the node metabolite pyruvate on the metabolic map (see below). In good agreement with such a connection, the activity of both PdhR [3] and FadR [20] is controlled by pyruvate as an effector ligand. To examine the influence of pyruvate on the reaction *in vivo* of fatty acid degradation, *E. coli* wild-type BW25113 was grown in the absence and presence of 3 mM pyruvate. Whole RNA from each culture was subjected to RT-qPCR assay. *E. coli* K-12 contains two pyruvate-sensing two-component systems (TCS), high-affinity BtsSR and low-affinity PyrSR [22, 23]. The inducible pyruvate importer BtsT is under the control of BtsSR, and another transporter YhjX is under the control of PyrSR. Expression levels of both *btsT* and *yhjX* increased 49.6±14.5- and 678.0±154.2-fold, respectively, by adding 3 mM pyruvate, in agreement with their induction by pyruvate-sensing PyrSR [23]. In the presence of pyruvate, both *aceE* and *ndh* increased to a level as high as that of the *pdhR* deletion mutant (Fig. 7) again in agreement with our previous observation [3]. In contrast, the expression of newly identified PdhR target genes for fatty acid degradation decreased 2- to 5-fold by addition of pyruvate, implying deactivation of PdhR by pyruvate with respect to fatty acid degradation. The binding activity of PdhR was identified on the *fadIJ* and *fadE* promoters by gSELEX (see Fig. 1, Table 1), but PdhR binding was also detected, albeit at low levels, at promoter regions of the *fadM* (thioesterase III), *fadL* (transporter for long-chain fatty acid) and *fadH* (2, 4-dienoyl-CoA reductase) genes, all involved in fatty acid degradation (for details see Fig. S1, available in the online version of this article). Expression of these genes was also decreased by addition of pyruvate.

**Fig. 8.**
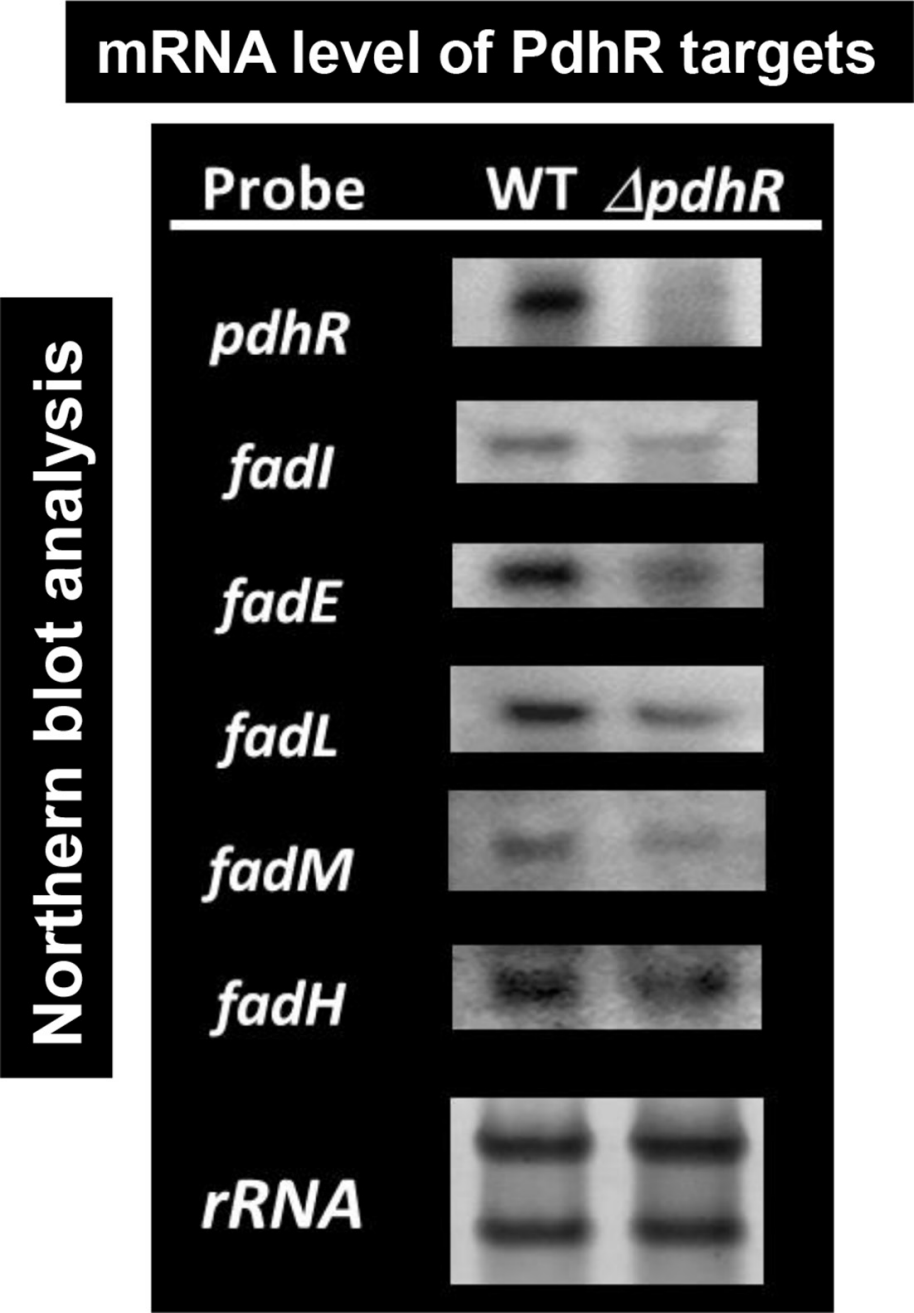
Northern blot analysis of mRNAs for fatty acid degradation. *E. coli* wild-type BW25113 and its *pdhR* mutant JW0109 were grown in M9 minimal medium with 0.2 % succinate. Total RNA was prepared at the exponential phase and directly subjected to Northern blot analysis under the standard conditions as described in the Methods. DIG-labelled hybridization probes are shown on the left side of each panel. The amounts of PdhR target mRNAs were measured as relative levels of 23S and 16S rRNAs stained with methylene blue.

To confirm the results of the RT-qPCR assay, we also performed Northern blot analysis with use of DIG-labelled probes. In the absence of PdhR, the mRNA level decreased for *fadI, fadE, fadL, fadM* and *fadH* (Fig. 8), supporting the notion that PdhR activates a set of genes for fatty acid degradation only in the absence of effector pyruvate. As noted above, the activation of the fatty acid degradation genes by PdhR could be lost in the presence of pyruvate, the effector ligand of RdhR.

## Discussion

### Novel regulatory role of PdhR: CCM

*E. coli* K-12 contains a total of about 300 TFs [12, 24]. We have been involved in a systematic search of regulatory targets for all these TFs by using gSELEX screening. Up to the present time, we have succeeded in listing the regulatory target promoters, genes and operons for about half of *E. coli* TFs (see the records in Ishihama *et al.* [12]; and also TEC database: https://shigen.nig.ac.jp/ecoli/tec/top/). One interesting finding is that the number of regulation targets is highly variable, ranging from single targets, referred to as single-target TFs [12], to more than 500, referred to as global TFs [12, 25]. For instance, 200–300 targets were identified for two global regulators of carbon source metabolism, CRP [cyclic AMP (cAMP) receptor protein] and Cra (catabolite repressor activator) [26, 27]. The majority of CRP targets are genes coding for the selective transport of carbon sources and the gluconeogenesis pathway, the TCA cycle, PDHc and aerobic respiration [26, 28, 29], and CRP activity depends on cAMP, the signal induced in the absence of glucose [30, 31]. In the case of Cra, the major target includes the genes coding for the enzymes involved in CCM, covering all the genes for the enzymes involved in glycolysis and metabolism downstream of glycolysis, including the TCA cycle and aerobic respiration [27, 32, 33], and Cra activity depends on d-fructose 1-phosphate and d-fructose 1,6-bisphosphate [34].

**Fig. 9.**
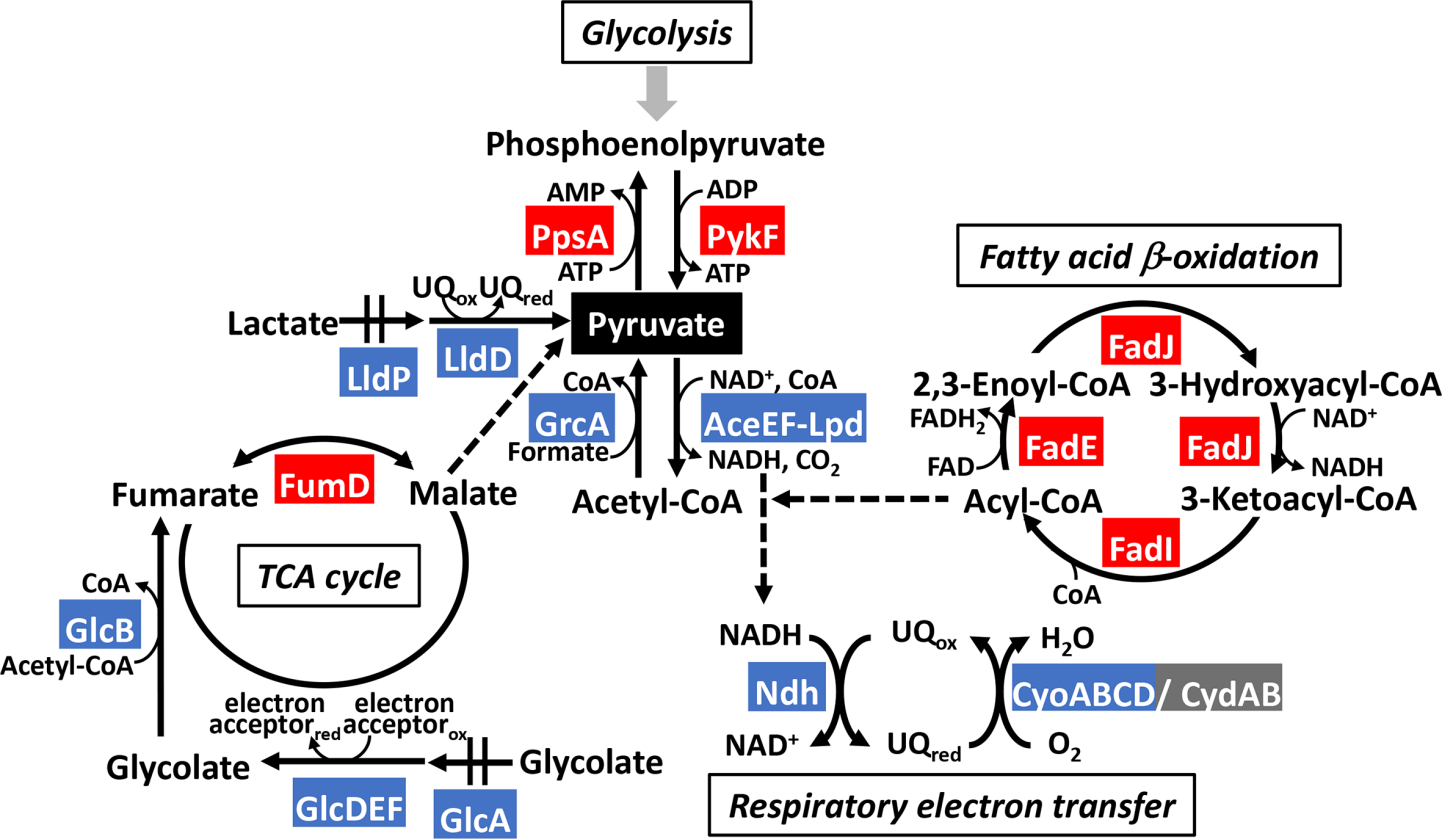
Model of the PdhR regulon. The whole set of regulatory targets of PdhR obtained in this study are aligned along the *E. coli* metabolism pathway. The PdhR targets include the genes involved in carbon source catabolism from PDH down to respiratory electron transport, lactate degradation, TCA cycle and β-oxidation of fatty acid degradation. The genes repressed by PdhR are shown in blue while the genes activated by PdhR are shown in red. The PdhR regulon members, *ppsA, pykF, lldP, lldD, fumD, fadE, fadI* and *fadJ,* were identified in this study.

The number of regulatory targets of PdhR related to carbon source metabolism was less than 20, and thus PdhR should be classified as a local regulator (Fig. 9) [25]. Of note is that the PdhR-regulated targets herein identified are also under the control of CRP and/or Cra, suggesting coordinated regulation between the global TFs (CRP and Cra) and the local TF (PdhR).

### Novel regulatory role of PdhR: fatty acid catabolism

In addition, a set of genes (*fadI, fadJ* and *fadE*) encoding the enzymes involved in fatty acid degradation were found to be the regulatory targets of PdhR (see Fig. 1, Table 1). The genes *fadM* (thioesterase III) and *fadH* (2, 4-dienoyl-CoA reductase) also involved in fatty acid degradation were found to be the regulatory targets of PdhR (Figs 7 and 8, Table 1). In the absence of favourable carbon sources, *E. coli* imports and utilizes fatty acids as carbon sources for generation of metabolic energy. Fatty acids are imported into *E. coli* cells through an outer membrane-associated channel (FadL) and inner membrane-associated long-chain-fatty-acid-CoA ligase (FadD), and then degraded via β-oxidation [19, 35] (Fig. 9). *E. coli* has two types of β-oxidation pathway: one operating by the enzymes FadA, FadB and FadE; and another by the enzymes FadI, FadJ and FadE. The latter pathway is preferred under anaerobic conditions. The differential utilization model of two pathways appears not only at the level of oxygen supply but also that of fatty acid species [19]. As carbon sources, fatty acids are degraded to produce NADH, FADH_2_ and acetyl CoA, which is then integrated into the TCA cycle for further generation of metabolic energy [14, 36] (Fig. 9). The final product acetyl-CoA of fatty acid degradation is then integrated into the main pathway of catabolism of carbon sources (Fig. 9).

Fatty acids are essential components of cell membranes and are important sources of metabolic energy in bacteria. Therefore, two pathways, fatty acid degradation and fatty acid biosynthesis, are controlled according to the availability of fatty acids to maintain membrane lipid homeostasis and the need for metabolic energy. For this switch control, a number of TFs are involved. GntR-family FadR plays a major role in the regulation of fatty acid degradation. All of these six genes under the control of PdhR are known to be repressed by FadR [37, 38]. The DNA-binding activity of FadR is inactivated after interaction with acyl-CoA, the cofactor of fatty acid metabolism [20]. Except for the *fadM* gene, the other five genes are repressed by ArcA (anoxic redox control), the response regulator of the TCS ArcAB that responds to change in the respiration state via monitoring the redox state of the ubiquinone and the menaquinone pool [39, 40]. Besides PdhR, FadR and ArcA, some other TFs are known in regulation of the *fad* regulon: OmpR for repression of *fadL* [41]; CRP for activation of *fadL* [26, 42] and *fadH* [26, 43]; and HipB for repression of *fadH* [44].

### Novel regulatory role of PdhR: motility control

One unexpected finding of gSELEX screening of PdhR targets is its involvement in regulation of the *fliAZ* operon (see Fig. 1, Table 1). The synthesis, assembly and function of the flagellar and chemotaxis system require the expression of more than 50 genes, which are divided into three temporally regulated transcriptional stages based on the hierarchy of expression order [15]. The *fliAZ* operon is the second-stage gene group in the hierarchy of flagellar formation. FliA (RpoF) controls the third-stage gene group [15, 17]. Using gSELEX screening, we have identified the total set of constitutive promoters that are recognized and transcribed by each of the RNA (RNA polymerase) holoenzymes alone in the absence of TFs. In the case of RpoF, 34–41 regulatory targets have been identified, including the stage 3 genes of flagella formation [45]. Here we found that RpoF-regulated synthesis of flagellar components is controlled by pyruvate-sensing PdhR. In the absence of PdhR, the expression level of the flagellar component increased (see Fig. 4), indicating a new model in which the level of flagella is controlled by the level of pyruvate.

### Pyruvate: the key node for metabolic homeostasis

Pyruvate, the terminal product of glycolysis, plays a key role in connecting a variety of metabolic pathways [7, 8]. Pyruvate forms a node between a number of metabolic pathways, including the TCA cycle, gluconeogenesis, fatty acid synthesis and amino acid synthesis. In response to stressful environmental conditions, pyruvate acts as a scavenger of reactive oxygen species.

The major role of pyruvate-sensing PdhR in carbon metabolism is to maintain the homeostasis of the intracellular pyruvate pool and acetyl-CoA depending on the species and level of carbon sources. PdhR also plays an important role in the metabolic reprogramming during growth switch from anaerobic to aerobic conditions [46]. Pyruvate acts as an allosteric effector for control of the activity of a set of glycolytic and gluconeogenetic enzymes such as isocitrate dehydrogenase (Icd) [47], ADP-glucose pyrophosphorylase (GlgC) [48] and fructose 1,6-bisphosphatase (Fbp) [49]. Pyruvate acts as an effector ligand for control of the activity of TFs, PdhR [3] and IclR (isocitrate lyase regulator) [50]. Taken together, the present study reveals more details about the key node role for pyruvate in *E. coli* metabolism.

## Methods

### Bacterial strains and plasmids

*Escherichia coli* K-12 W3110 type-A [51] was used as the DNA source for the construction of PdhR expression plasmid. *E. coli* K-12 W3110 type-A genome was also used for construction of the DNA library for SELEX screening. *E. coli* DH5α was used for plasmid amplification. *E. coli* BL21 (DE3) was used for PdhR expression. *E. coli* BW25113 [52], its *pdhR* knock-out mutant JW0109 and *fadR* knock-out mutant JW1176 [53] were obtained from the *E. coli* Stock Centre (National Bio-Resource Centre, Japan). The PdhR expression plasmid and the control empty plasmid pCA24N*Δgfp* were obtained from the National BioResource Project (National Institute of Genetics, Japan). JW0109 lacking a kanamycin marker was constructed in this study using the temperature-sensitive plasmid pCP20 carrying FLP recombinase [54]. Plasmid pPET21 was used for construction of the PdhR expression plasmid pPdhR for PdhR protein purification. Cells were grown in M9 minimal medium supplemented with 0.2 % succinate at 37 °C with constant shaking at 150 r.p.m. When necessary, kanamycin at 20 μg ml^−1^ was added to the medium. Cell growth was monitored by measuring the turbidity at 600 nm.

### Purification of PdhR protein

Plasmid pPdhR for expression and purification of PdhR was constructed according to standard procedures [3]. In brief, PdhR coding sequences were PCR-purified using the *E. coli* K-12 W3110 genome DNA as a template, and inserted into pET21a (+) vector (Novagen) between *Nde*I and *Not*I sites, leading to construction of pPdhR. The expression plasmid pPdhR was transformed into *E. coli* BL21 (DE3). The transformants were grown in LB medium and PdhR was expressed by adding IPTG in the middle of exponential phase growth. PdhR protein was purified by the affinity purification procedure with use of an Ni-nitrilotriacetic acid (NTA) agarose column. The affinity-purified PdhR protein was stored frozen in storage buffer at −80 °C until use. Protein purity was more than 95 % as checked by SDS-PAGE.

gSELEX screening of PdhR-binding sequences was carried out as previously described [9, 10]. In brief, a mixture of DNA fragments of the *E. coli* K-12 W3110 genome was prepared after sonication of purified genomic DNA, and cloned into a multi-copy plasmid pBR322. In each gSELEX screening, the DNA mixture was regenerated by PCR. For gSELEX screening, 5 pmol of the mixture of DNA fragments and 10 pmol PdhR were mixed in a binding buffer (10 mM Tris-HCl, pH 7.8 at 4 °C, 3 mM magnesium acetate, 150 mM NaCl and 1.25 mg ml^−1^ BSA) with or without 1 mM sodium pyruvate. The SELEX cycle was repeated five times for enrichment of PdhR-binding sequences. Mapping of SELEX fragments along the *E. coli* genome was also performed by the gSELEX-chip system by using a 43‌450-feature DNA microarray (Oxford Gene Technology). The gSELEX sample obtained with use of PdhR was labelled with Cy3, while another SELEX sample obtained in the absence of PdhR addition was labelled with Cy5. After hybridization of samples to the DNA tiling array, the Cy3/Cy5 ratio was measured and the peaks of scanned patterns were plotted against the positions of DNA probes along the *E. coli* K-12 genome.

### Gel shift assay

The gel shift assay was performed according to standard procedures [55]. Probes of the PdhR-binding target sequences were generated by PCR amplification using a pair of primers (Table S1a) and Ex Taq DNA polymerase (Takara). For gel shift assays, a mixture of each probe and PdhR was incubated at 37 °C for 30 min in the gel shift buffer. After addition of a DNA loading solution, the mixture was directly subjected to PAGE. DNA in gels was stained by GelRed (Biotium) and detected using LuminoGraph I (Atto).

### Consensus sequence analysis

To analyse the PdhR binding sequence, a set of PdhR binding sequence identified by gSELEX-chip was analysed by using the program dminda 2.0 [56]. Sequences were aligned and a consensus sequence logo was created by weblogo (http://weblogo.berkeley.edu/logo.cgi).

### RT-qPCR analysis

RT-qPCR analysis was performed according to standard procedures [57]. *E. coli* cells were inoculated in M9 minimal medium supplemented with 0.2 % succinate at 37 °C under aeration with constant shaking at 150 r.p.m. until an OD_600_ of 0.3, then total RNAs were extracted. Total RNAs were transcribed to cDNA with random primers using the THUNDERBIRD SYBR qPCR RT Set (TOYOBO). Quantitative PCR (qPCR) was conducted using THUNDERBIRD SYBR qPCR Mix (TOYOBO) and was performed using the LightCycler 480 system (Roche). Pairs of primers used are described in Table S1b. The cDNA templates were serially diluted four-fold and used in the qPCR assays. The qPCR mixtures, each containing 10 µl of THUNDERBIRD SYBR qPCR Mix (TOYOBO), 1 µl of each primer (5 µM stock), 7 µl of water and 1 µl of cDNA, were ampliﬁed under the following thermal cycle conditions: 95 °C treatment for 2 min; 45 cycles of 10 s at 95 °C and 20 s at 55 °C; and incubation for 20 s at 72 °C. The expression levels of 16S rRNA were used for normalization of the levels of test samples, and the relative expression levels were quantiﬁed using Relative Quantification software provided by Roche. The results presented are averages of the results from three experiments.

### Motility assay

To observe cell motility, *E. coli* cells were inoculated on the swarm plate based on M9 minimal medium supplemented with 0.2 % succinate and 0.25 % Bacto-agar. The swarm plate was inoculated with a stab of approximately 10^6^ cells and incubated at 37 °C for 24 h. After 24 h, the plate was imaged and colony diameter was measured as the motility zone. The results presented are averages of the results from five experiments.

### Fatty acid analysis

Fatty acid extraction was performed according to Lu *et al.* and Liu *et al.* with some modifications [58, 59]. To compare the amount of free fatty acids and their composition, *E. coli* cells were inoculated in 100 ml of M9 minimal medium supplemented with 0.2 % succinate at 37 °C under aeration with constant shaking at 120 r.p.m. until an OD_600_ of 0.4. The fatty acid content of inoculated cells was extracted by chloroform/methanol (1 : 2, v/v), and again by chloroform/methanol (1 : 2, v/v) with 1 % KCl. Chloroform with 1 % KCl was added to the resulting mixture and vortexed, then centrifuged. The upper layer was collected and dried with nitrogen gas for 45 min. Then chloroform/methanol (2 : 1, v/v) was used to resuspend the sample before loading onto a TLC plate. The TLC plate was developed with a solvent including hexane/dietyl ester/acetic acid (160:40:4; by vol.), and dried. Free fatty acids were detected and sprayed with 0.001 % primuline in 80 % (v/v) acetone and exposed to UV light. The spot of free fatty acid was scraped and added to 100 µl of 1 mM pentadecanoic acid (C15 : 0) and 500 µl of 5 % (v/v) HCl in methanol, and incubated at 85 °C for 1 h. After addition of 500 µl hexane, the sample was vortexed and centrifuged. The supernatant was transferred into a new tube and dried with nitrogen gas. The free fatty acid was resuspended with 60 µl hexane and subjected to GC-FID analysis. GC-FID analysis was performed according to Imamura *et al.* with the gas chromatograph (Shimazu GC-2014) equipped with a flame ionization detector, along with an ULBON HR-SS-10 capillary column (25 m×0.25 mm) [60]. Helium was used as a carrier gas at a flow rate of 20 cm s^–1^. The detector temperature was 250 °C. The column temperature, after an initial isothermal period of 5 min at 140 °C, was increased to 200 °C at a rate of 4 °C min^−1^ and maintained for 10 min after reaching 200 °C. The results presented are averages of the results from three experiments, and differences between strains were confirmed by tests of significance.

### Northern blot analysis

Total RNAs were extracted from exponentially growing *E. coli* cells (OD_600_ of 0.3–0.4) by using ISOGEN solution (Nippon gene). RNA purity was checked by electrophoresis on a 1.5 % agarose gel in the presence of formaldehyde followed by staining with GelRed. Northern blot analysis was performed essentially as described previously [61]. DIG-labelled probes were prepared by PCR amplification using W3110 genomic DNA (50 ng) as template, a pair of primers (Table S1c), DIG-11-dUTP (Roche) and dNTP as substrates, gene-specific forward and reverse primers, and Ex Taq DNA polymerase. Total RNAs (3 µg) were incubated in formaldehyde-MOPS gel-loading buffer for 10 min at 65 °C for denaturation, subjected to electrophoresis on formaldehyde-containing 1.5 % agarose gel, and then transferred to a nylon membrane (Roche). Hybridization was performed with the DIG easy Hyb system (Roche) at 50 °C overnight with a DIG-labelled probe. For detection of the DIG-labelled probe, the membrane was treated with anti-DIG-AP Fab fragments and CDP-Star (Roche), and the image was scanned with LuminoGraph I (Atto).

## Supplementary Data

Supplementary material 1

Supplementary material 2
