# Supplementary figures and images for: Expanded roles of pyruvate-sensing PdhR in transcription regulation of the Escherichia coli K-12 genome: fatty acid catabolism and cell motility
Source: Microb Genom. 2020 Sep 25;6(10):mgen000442. doi: 10.1099/mgen.0.000442 (PMC7660256; doi:10.1099/mgen.0.000442)

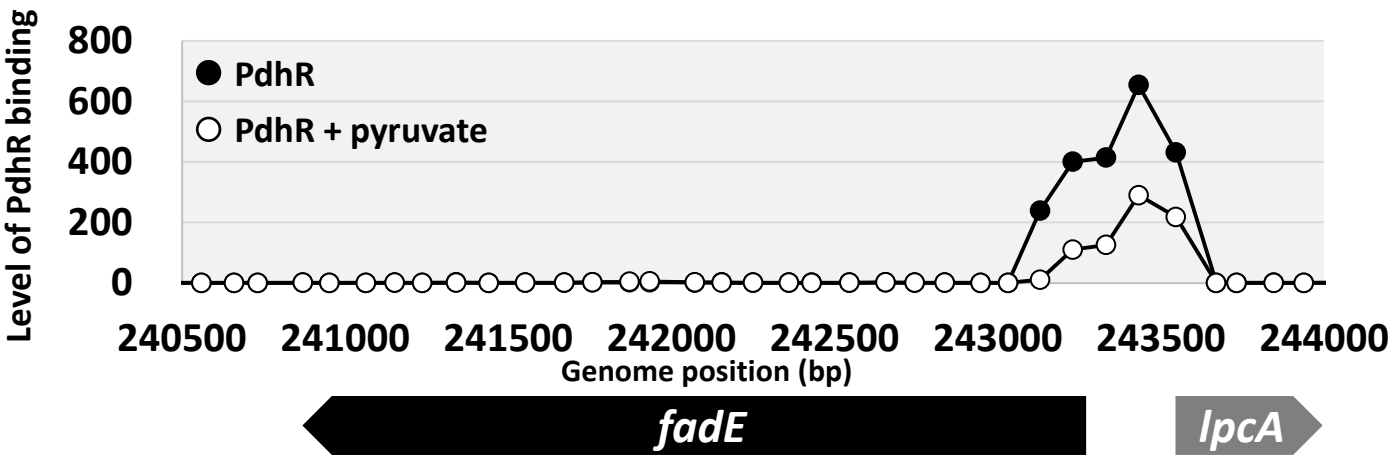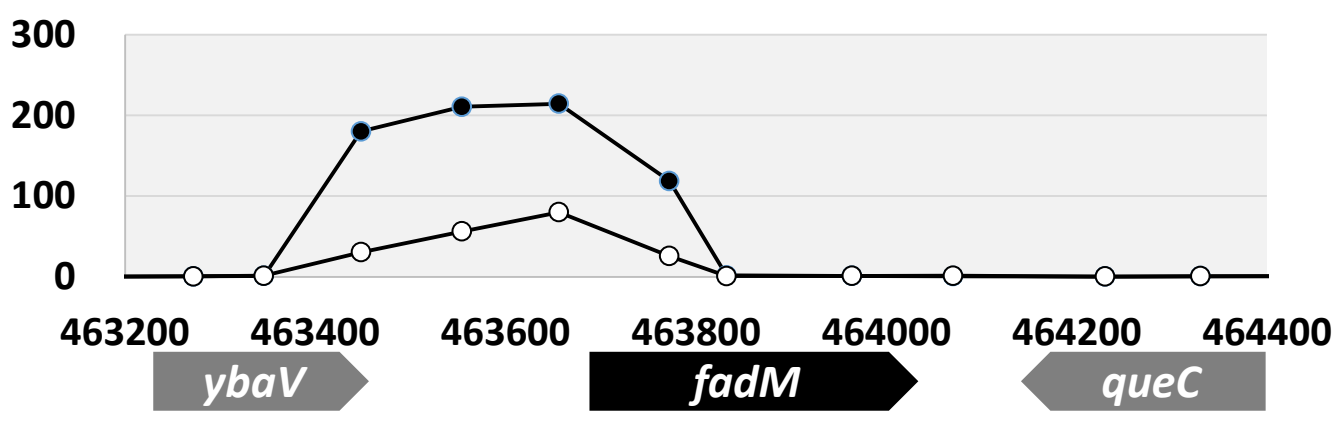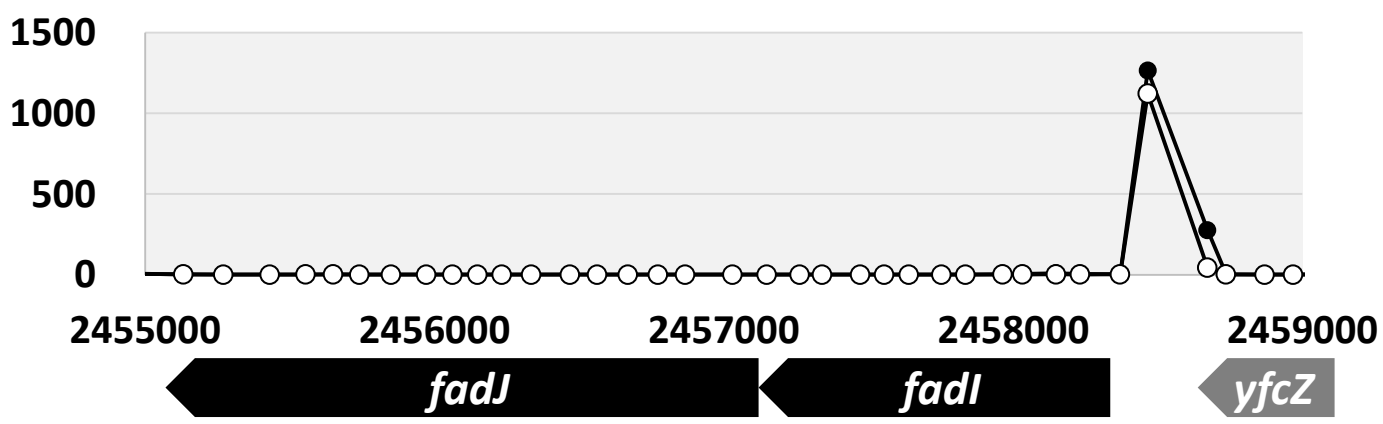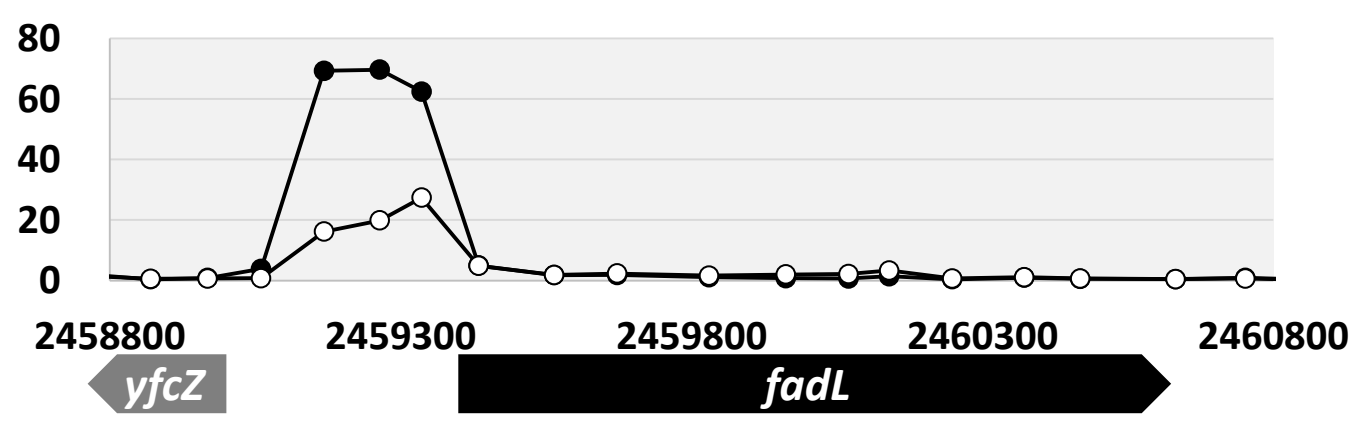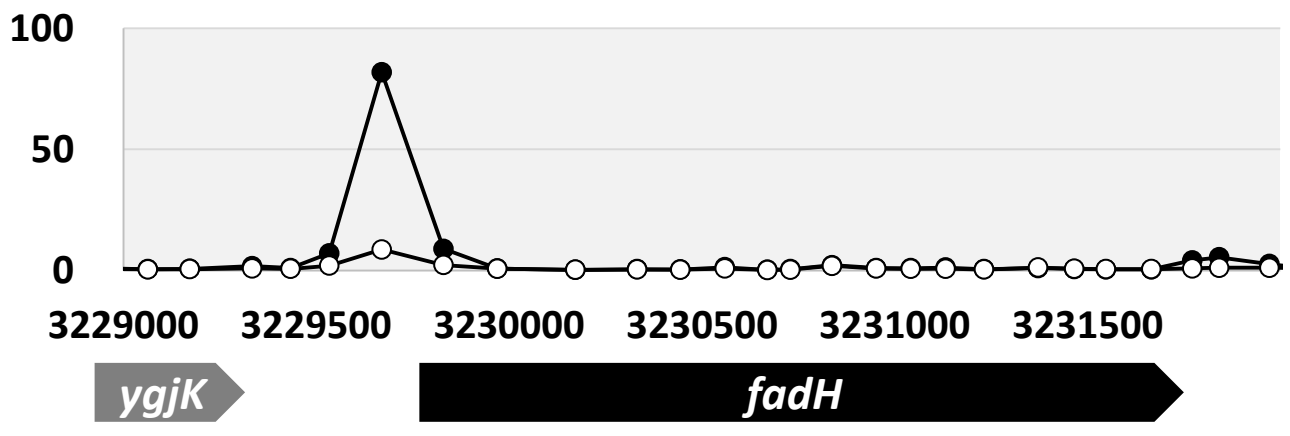

Supplement: Supplementary material 1 [file mgen-6-442-s001.pdf]
